# A Mechanistic End-to-End Concussion Model That Translates Head Kinematics to Neurologic Injury

**DOI:** 10.3389/fneur.2017.00269

**Published:** 2017-06-15

**Authors:** Laurel J. Ng, Vladislav Volman, Melissa M. Gibbons, Pi Phohomsiri, Jianxia Cui, Darrell J. Swenson, James H. Stuhmiller

**Affiliations:** ^1^Simulation Engineering and Testing, L-3 Applied Technologies, Inc., San Diego, CA, United States; ^2^Cardiac Rhythm and Heart Failure Numerical Modeling, Medtronic, Mounds View, MN, United States

**Keywords:** concussion mechanism, mTBI, internal dose, node of Ranvier, axon injury, axonal dysfunction, kinematic correlates, dose-response

## Abstract

Past concussion studies have focused on understanding the injury processes occurring on discrete length scales (e.g., tissue-level stresses and strains, cell-level stresses and strains, or injury-induced cellular pathology). A comprehensive approach that connects all length scales and relates measurable macroscopic parameters to neurological outcomes is the first step toward rationally unraveling the complexity of this multi-scale system, for better guidance of future research. This paper describes the development of the first quantitative end-to-end (E2E) multi-scale model that links gross head motion to neurological injury by integrating fundamental elements of tissue and cellular mechanical response with axonal dysfunction. The model quantifies axonal stretch (i.e., tension) injury in the corpus callosum, with axonal functionality parameterized in terms of axonal signaling. An internal injury correlate is obtained by calculating a neurological injury measure (the average reduction in the axonal signal amplitude) over the corpus callosum. By using a neurologically based quantity rather than externally measured head kinematics, the E2E model is able to unify concussion data across a range of exposure conditions and species with greater sensitivity and specificity than correlates based on external measures. In addition, this model quantitatively links injury of the corpus callosum to observed specific neurobehavioral outcomes that reflect clinical measures of mild traumatic brain injury. This comprehensive modeling framework provides a basis for the systematic improvement and expansion of this mechanistic-based understanding, including widening the range of neurological injury estimation, improving concussion risk correlates, guiding the design of protective equipment, and setting safety standards.

## Introduction

Concussion is the result of a cascade of events with violent head motion as the initiator. Head kinematics has previously served as the basis of concussion correlates because it yields readily measurable external parameters, such as peak linear or rotational head acceleration, which are assumed to be related to a tissue response and injury. These correlates, however, are usually limited in applicability to the conditions in which the data are collected (1–6), and thus are restrictive in nature. Other correlates have been developed from small primate kinematic data, but require empirical scaling for application to humans (7). External correlates describe the input or exposure conditions that drive the injury outcomes but do not explain why an injury results. However, an internal injury correlate is a fundamental quantity of injury that is independent of exposure conditions, boundary conditions, and species. Therefore, correlates developed from internal injury measures are applicable under a broad range of conditions, do not require scaling, and provide insight into the injury mechanism. The development of a robust injury correlate relies on the quantification of an internal injury measure, requiring a mechanistic understanding of the entire injury pathway.

Years of research into concussion have produced a considerable volume of knowledge regarding injury mechanisms on discrete scales (e.g., tissue, cellular) that result in a cascade of underlying pathophysiological responses (e.g., neuronal depolarization, ionic imbalance, impaired axonal function). There is a general understanding of how these processes combine to translate head motion into mechanical disruption and neurophysiological aberrations on the cellular level, which can then be linked to concussive outcomes; however, no quantitative models exist that connect these multi-scale injury mechanisms into an integrated end-to-end (E2E) model.

Concussion starts with loading to the head (e.g., from impact or blast exposure), which results in the head inertial acceleration and/or load transmission through the head or torso that in turn yields cellular-level neurological injury. Head loadings that produce violent head motion result in localized dynamic stresses and strains throughout brain tissue (8, 9). Several finite element model (FEM) studies have quantified threshold stresses and strains in localized brain regions (such as the corpus callosum, the midbrain, and the brainstem) that are associated with concussive outcomes (3, 6, 10–14). These threshold studies utilized the notion of diffuse axonal injury, in which bundles of axonal fibers throughout the brain are affected, likely as a result of loading-related axonal strains (6, 15–17). These studies indicate that large maximal principal strains and strain rates are likely to occur in the corpus callosum (12), a prominent brain structure that is comprised of a bundle of myelinated axons that mediate information exchange between the left and right hemispheres.

Diffusion tensor imaging (DTI) has provided further evidence linking concussion to injury of the white matter tracts, including the corpus callosum (12, 18, 19). DTI quantifies molecular diffusion of water in the brain tissue and provides a structural map of white matter directionality throughout the brain (20). DTI studies revealed reduced fractional anisotropy and decreased mean diffusivity in the corpus callosum of concussed individuals, implying that axonal integrity and functionality are affected in concussion (21). Together with FEM analyses, injury thresholds based on head kinematics were developed (6, 15). It is not surprising that myelinated axons are affected by concussive head loadings; long sections of relatively viscous myelin that are interrupted by short, relatively elastic non-myelinated regions (nodes of Ranvier) are likely to make the nodes particularly susceptible to strain concentration, especially at high strain rates. Indeed, severe elongation of nodes of Ranvier has been observed in histological sections of axons in tension (22).

Physical stretching of nodes of Ranvier beyond a critical threshold can lead to injury on the subcellular level. Nodal injury can strongly affect axonal signal propagation, and this can be understood by considering the nodal ultra-structure. Nodes of Ranvier have a high density of voltage-gated sodium (Na^+^) channels, which play a critical role in the regeneration and propagation of action potentials along the axon. Animal models of axonal stretch injury have demonstrated that high axonal strains result in strain-induced injury of nodal tetrodotoxin-sensitive voltage-gated Na^+^ channels, manifested as a stretch-magnitude dependent shift in the channels’ activation and inactivation voltages (23), which in turn triggers a cascade of ion redistribution events (such as influx of calcium ions) (24), culminating in axonal signaling dysfunction and potentially in axonal degeneration (25). These observations suggest that axonal dysfunction can be used as an internal injury metric of concussion.

The objective of this paper is to translate these fundamental processes that result in brain injury into a quantitative, mechanistic-based E2E concussion model that links head kinematics to neurological injury following axonal tensile stretching and subsequent damage to the nodes of Ranvier. By modeling the internal injury, this model can be applied to describe a range of exposure types and conditions. More importantly, a mechanistic-based model has a much broader application beyond estimating concussion risk; it can be used to guide development of protective equipment, help set safety standards, and improve current and future monitoring technologies. Furthermore, once the foundation for linking head motion to the mechanism of injury at the cellular level has been established, extensions can be made to assess more complicated outcomes, such as neurobehavioral sequelae, and more subtle mechanisms of action, such as accumulation of subthreshold head injuries.

## Materials and Methods

**Figure 1:**
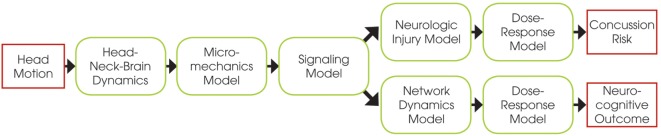
Schematic of the end-to-end model, showing the relations between the different component models.

The E2E concussion model is comprised of a multi-scale set of validated component models that link head kinematics to axonal signaling dysfunction in the corpus callosum and altered cortical dynamics (Figure 1). The E2E model starts with input of the head kinematics into a head FEM. The FEM results are processed to calculate transient axonal strains in the elements of the corpus callosum, which are then translated into localized axonal strains and injury of the axonal nodes of Ranvier *via* a micromechanical model of the myelinated axon. This physical injury is captured as signaling dysfunction by a biophysical signaling model that relates injury of nodal tetrodotoxin-sensitive voltage-gated Na^+^ channels to injury-induced changes in the amplitude and latency of action potentials propagating along the injured axons. From this, a neurologic injury measure (NIM) is calculated by volume-weighted averaging of signal dysfunction over all elements in the corpus callosum. The NIM serves as the internal injury correlate based on which a dose–response curve is derived. A network model of spiking neurons, capturing intra- and inter-hemispheric cortical dynamics modulated by the corpus callosum, simulates changes in the communication dynamics based upon the corpus callosum injury severity.

### Human and Non-Human Primate (NHP) Head FEMs

**Figure 2:**
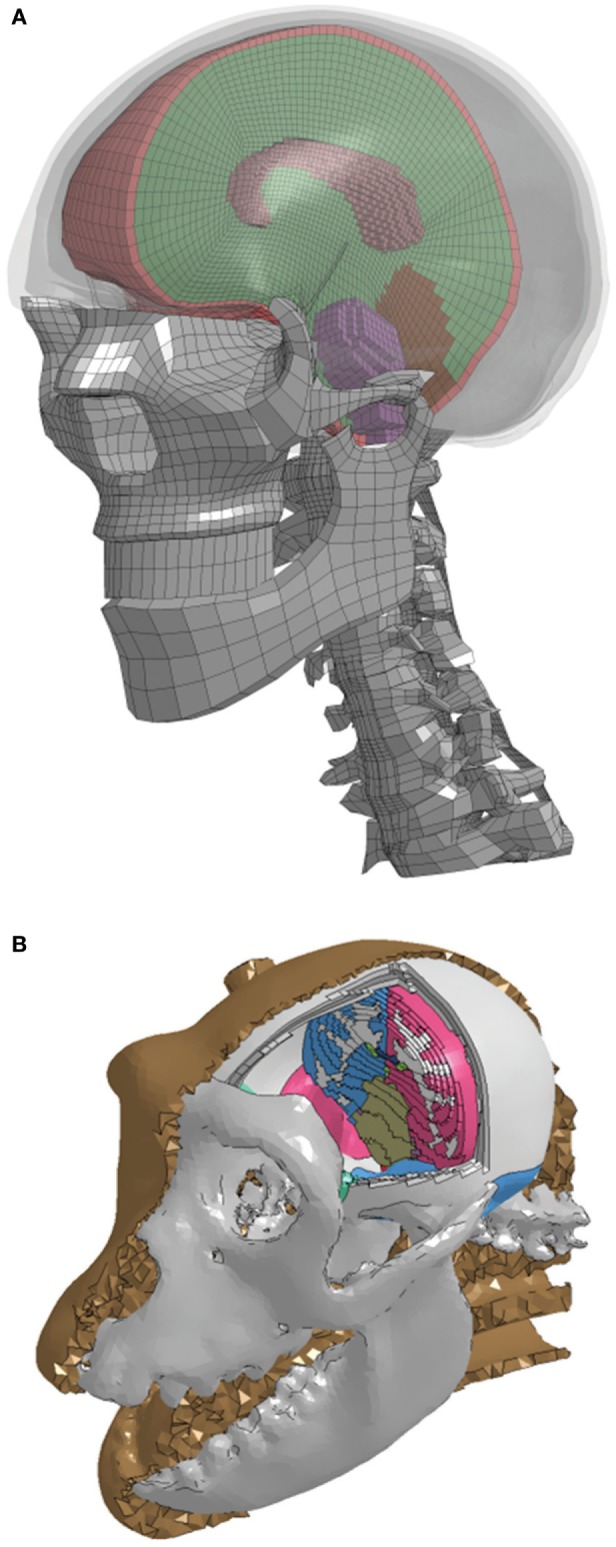
A cut view of the high-resolution segmented brain mesh of the finite element model for the **(A)** human and **(B)** non-human primate.

Human and NHP head FEMs coupled with DTI data were developed to translate head kinematics into transient strains in the axial direction of the corpus callosum myelinated axons. A detailed FEM of the human head was constructed from computed tomography (CT) data acquired from the Visible Human Project (26), with the hexahedral brain mesh segmented into the major anatomical components (right and left cerebri, right and left cerebelli, corpus callosum, and brainstem) using the Zygote anatomical dataset,^1^ as shown in Figure 2. The tentorium cerebelli and falx cerebri were modeled as shell layers defined by the boundary nodes between the cerebrum and cerebellum and between the cerebral hemispheres, respectively. The outermost layer of solid elements in the brain mesh was separated to represent the dura mater and cerebrospinal fluid (CSF). The outer surface of the cerebrum was separated into a single shell layer to represent the arachnoid and pia mater, which are very thin, stiff membranes, providing a layer of protection around the cerebrum. While the FEMs include the geometry of the facial bones and cervical spine, these structures did not play a role in the current simulations because measured head kinematics were applied to a rigid skull. Skull features and properties become necessary when simulating an impact or blast event to the head, and the resulting head motion is calculated.

Tied contact was enforced at the interface between the inner surface of the skull and the outer surface of the dura–CSF, and a frictionless sliding (no separation) contact was enforced at the interface between the inner surface of the dura–CSF and the outer surface of the pia–cerebrum (27). Additionally, the outermost nodes of the tentorium cerebelli and falx cerebri shell components were tied to the dura, to model the attachment of these membranes to the skull. The brain tissue material properties were bounded by values identified in literature (28). The tissue was modeled as a nearly incompressible isotropic viscoelastic material with initial material properties based upon the 2001 version of the Wayne State University Head Injury Model (29). The dura–CSF, pia–arachnoid mater, falx cerebri, and tentorium cerebelli components were modeled as elastic, with property values taken from Ref. (11). The material parameters were then calibrated to reflect dynamic deformation captured by cadaver impact studies (8). The model material parameters are listed in Table 1. The model was validated against simulation of four decelerative impacts in which brain displacement data were measured (9).

**Table 1:** FE model material properties.

		Human FE model					Monkey FE model				
Material	Constitutive law	*ρ* (kg/m^3^)	*K* (GPa)	*G*_0_ (Pa)	*G*_∞_ (Pa)	*β* (1/s)	*ρ* (kg/m^3^)	*K* (GPa)	*G*_0_ (Pa)	*G*_∞_ (Pa)	*β* (1/s)
Corpus Callosum	Viscoelastic	1,040	2.19	5,000	500	80	1,040	2.19	18,500	6,700	100
Cerebrum	Viscoelastic	1,040	2.19	4,000	400	80	1,040	2.19	10,300	3,700	100
Cerebellum	Viscoelastic	1,040	2.19	3,000	300	80	1,040	2.19	10,300	3,700	100
Brainstem	Viscoelastic	1,040	2.19	6,000	600	80	1,040	2.19	18,500	6,700	100

**Material**	**Constitutive law**	***ρ* (kg/m^3^)**	***E* (MPa)**	***ν***	***t* (mm)**		***ρ* (kg/m^3^)**	***E* (MPa)**	***ν***	***t* (mm)**	

Dura	Elastic	1,130	31.5	0.45	–		1,040	40	0.45	–	
Falx	Elastic	1,140	31.5	0.45	1		1,040	4,000	0.45	1	
Tentorium	Elastic	1,140	31.5	0.45	1		1,040	4,000	0.45	1	
Pia-arachnoid	Elastic	1,140	6	0.45	1		1,040	12.5	0.45	1	

The NHP head FEM skull mesh was constructed from high-resolution CT scans of a rhesus macaque from the Primate Research Institute of Kyoto University. The hexahedral brain mesh was segmented to represent different regions of the brain based on a detailed rhesus macaque brain atlas taken from the INIA19 Primate Atlas found in the Neuroimaging Informatics Tools and Resources Clearinghouse (NITRC) database.^2^ These regions include the left and right cerebri (gray and white matter), left and right cerebelli (gray and white matter), medulla oblongata, pons, midbrain, corpus callosum, and ventricles. The treatment of the meninges and contact definitions were the same as in the human head FEM. A majority of the material properties were taken from literature (30). Those not available in the literature were taken from the human head FEM. The model material parameters are listed in Table 1.

Axonal strain in the corpus callosum was calculated by adapting a post-processing technique proposed by Chatelin et al. (31), rather than including the direction of white matter tracts within the constitutive models in the FEM. Wright et al. (32) found that the axonal strain response of an anisotropic white matter constitutive model can be fairly well-represented by an isotropic constitutive model when the continuum strain tensor is projected into the axonal axial direction using the DTI data. It was determined to be an acceptable alternative to the increased complexity and computational cost associated with incorporation of fiber orientation into the FEM, with both methods resulting in similar loci of high axonal strain (33). In order to transform the anatomical information from the DTI voxels to the finite elements, the DTI atlas was rigidly registered to the FEM brain. The average primary axonal axial direction was then calculated in each element of the corpus callosum component of the FEM. The average size of the corpus callosum elements was on the same order or slightly larger than the size of the DTI voxels; thus, a simple average of the primary axonal vectors from the voxels contained in each element was calculated. All average element axonal vectors were then normalized to ensure no influence of vector magnitude. The anatomical information about the primary axonal directions in human white matter came from the DTI data contained in the Illinois Institute of Technology Human Brain Atlas (V. 3) (34); in NHP, white matter came from the rhesus DTI atlas from the NITRC database.

### Axon Micromechanics Model

A micromechanics model of a myelinated axon was developed to translate the time histories of axonal strains of each element calculated by the FEMs into localized strains at the nodes of Ranvier. The micromechanics model had two major components: (1) a bare axon without myelin and (2) myelin.

The model of the underlying bare axon in the internodal and nodal regions followed the formulation of a spring in series with a Voigt element, as suggested by Dennerll et al. (35). The elastic moduli of the spring and Voigt elements (*E*_1_ and *E*_2_, respectively) and kinematic viscosity (η_1_) were calculated using

(1) $$E_{1}=\frac{k_{1}L}{A},\;E_{2}=\frac{k_{2}L}{A},\;\eta_{1}=\frac{\gamma_{1}A}{L}$$

where *k*_1_, *k*_2_ are the spring constants, γ_1_ is the viscous damping coefficient of the dashpot, *L* is the length of the section, and *A* is the corresponding cross-sectional area. *E*_1_, *E*_2_, and η_1_ were calculated to be 19.9 kPa, 0.42 kPa, and 2.256 MPa/s, respectively. The material properties of the springs and dashpot were based upon dorsal root ganglion neurite properties with length (*L*) and cross-sectional area (*A*) estimated from Dennerll et al. (35). For simplicity, we assumed a homogeneous bare axon; thus, the elastic moduli and viscosity were considered to be constant and geometry-independent.

Owing to its significant cholesterol content, the viscoelastic properties of myelin are likely to be quite different from those of a regular neural bilayer (35). Because the myelin consists of lipid and proteins, it was assumed that the myelin is viscoelastic, and the Maxwell material has been chosen to model it. To the best of our knowledge, the mechanical properties of myelin have not yet been characterized; thus, the values of the elastic modulus of the spring (*E*_3_) and the viscosity of the damper (η_3_) were parameterized to dynamic axonal stretching experiments of Singh et al. (36) and Rickett et al. (37) with an *E*_3_ of 50 kPa and η_3_ of 1 kPa/s.

**Figure 3:**
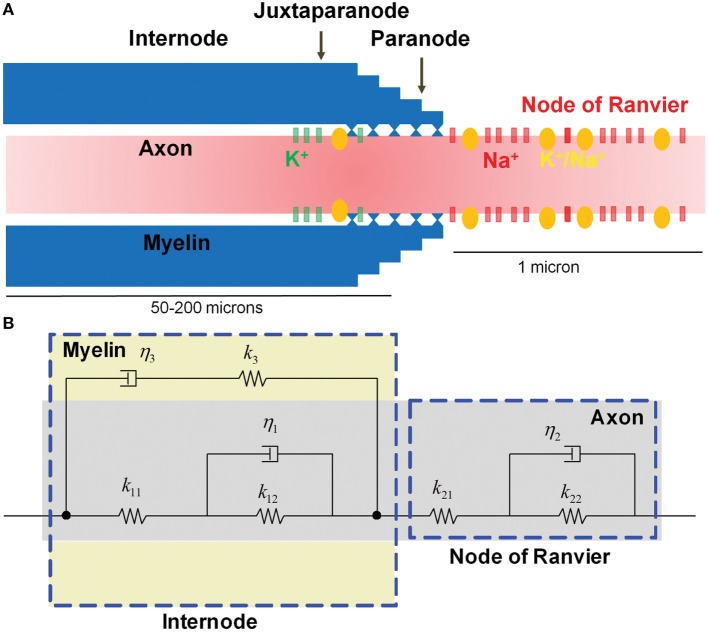
Microstructure of a myelinated axon. **(A)** The node of Ranvier contains a high concentration of voltage-gated Na^+^ channels that are injured as a result of physical stretching. **(B)** The micromechanical behavior is modeled as viscoelastic, with the myelin in the internode region exhibiting more viscous behavior compared with the relatively elastic axon.

Assembly of the bare axon with the myelin formed the myelinated axon model, which is illustrated in Figure 3. The complete myelinated axon model was composed of the myelinated internodal region and the unmyelinated node of Ranvier. Its spring and viscous damping constants were calculated using

(2) $$k_{11}=\frac{E_{1}A_{\text{internode}}}{L_{\text{internode}}},\;k_{12}=\frac{E_{2}A_{\text{internode}}}{L_{\text{internode}}}\;,\;\gamma_{1}=\frac{\eta_{1}A_{\text{internode}}}{L_{\text{internode}}}$$

(3) $$k_{21}=\frac{E_{1}A_{\text{node}}}{L_{\text{node}}},\;k_{22}=\frac{E_{2}A_{\text{node}}}{L_{\text{node}}},\;\gamma_{2}=\frac{\eta_{1}A_{\text{node}}}{L_{\text{node}}}$$

(4) $$k_{31}=\frac{E_{3}A_{\text{myelin}}}{L_{\text{internode}}},\;\gamma_{3}=\frac{\eta_{3}A_{\text{myelin}}}{L_{\text{internode}}}$$

where *L*_node_ and *L*_internode_ are the nodal and internodal lengths, respectively, and *A*_node_, *A*_internode_, *A*_myelin_ are the cross-sectional areas of the node, internode without myelin, and myelin, respectively. Given the inherent material differences between the myelinated region and the nodes of Ranvier, strains along the internode and the node of Ranvier are likely to depend on axonal strain and strain rate.

### Biophysical Signaling Model

A biophysical signaling model was developed to translate localized physical injury of the axon to functional decrements, using the NEURON simulator package (38). The biophysical model simulated action potential generation in the axonal initial segment, and its saltatory propagation along the myelinated axon, by considering a realistic ultra-structural axonal organization and distribution of biochemical mechanisms. Experimental evidence suggests a link between stretch injury of tetrodotoxin-sensitive voltage-gated Na^+^ channels (Nav1.6) and nodal excitability, which critically determines the function of myelinated axons (39, 40). Stretch injury has been shown to produce a leftward (toward more negative values) shift of the activation/inactivation voltages of these channels, in a manner that depends on the severity of stretching (23).

The electrochemical kinetics of action potential propagation were implemented using the NEURON simulator package (38). A one-dimensional multi-compartmental cable model with cylindrical geometry was used for describing the myelinated axon. The model myelinated axon consisted of a series of interconnected compartments, with compartmental properties matched to the known biophysical parameters for different axonal segments (nodes, paranodes connecting to nodes and juxtaparanodes, juxtaparanodes connecting to paranodes and internodes, and internodes connecting to juxtaparanodes) (25, 41, 42) (Figure 3A). The axon was ~10 mm long (although the length could be easily adjusted), with 100 fully myelinated internodal segments (length, 80 μm), interspersed by 101 non-myelinated nodes of Ranvier (length, 1 μm). On both flanks of each internode, myelinated juxtaparanodal compartments (length, 10 μm), housing voltage-gated potassium (K^+^) channels, bridged the internodes to the nodes of Ranvier *via* partially myelinated paranode compartments. At one of the model axon’s edges, the ultimate node of Ranvier was connected to a 5-μm-long non-myelinated axonal initial segment that contained a high density of voltage-gated Na^+^ and K^+^ channels, as well as Na^+^–K^+^ pumps. In this initial segment, channel kinetics were the same as for nodal and juxtaparanodal compartments (as described below).

For each compartment, the dynamics of the membrane potential, *V*_m_, were given by the following general equation

(5) $$C_{\text{m}}\frac{dV_{\text{m}}}{\mathrm{dt}}=-\sum I_{\text{Na}}-\sum I_{\text{K}}-I_{\text{L}}$$

where *C*_m_ is the compartmental membrane capacitance, Σ*I*_Na_ and Σ*I*_K_ are the sums of all Na^+^ and K^+^ currents for that same compartment, respectively, and *I*_L_ is the non-specific leak current for that same compartment. Membrane capacitance and resistance were set according to the level of compartment myelination (25). Voltage-gated Na^+^ channels were incorporated into nodes of Ranvier, while the presence of voltage-gated K^+^ channels were constrained to myelinated juxtaparanodes (43). Kinetics of nodal Na^+^ channels were modeled as follows, with the coupled left shift (CLS) injury modeled as in Boucher et al. (44), and derived from the original experimental data in Wang et al. (23).

Current due to voltage-gated Na^+^ channels, *I*_Na_, was modeled as

(6) $$I_{\text{Na}}={\tilde{g}}_{\text{Na}}\left[fm_{\Delta V}^{3}h_{\Delta V}+\left(1-f\right)m_{0}^{3}h_{0}\right]\left(V_{\text{m}}-E_{\text{Na}}\right)$$

where *E*_Na_ is the electrochemical-gradient dependent Nernst potential of Na^+^ ions, ${\tilde{g}}_{\text{Na}}$ is the single Na^+^ channel conductance, *f* is the fraction of injured channels per node, *m* and *h* are the Hodgkin–Huxley type Na^+^ channel activation and inactivation variables, respectively, and the parameter ΔV is the CLS, quantifying the nodal stretch injury.

Current due to voltage-gated K^+^ channels, *I*_K_, was modeled as

(7) $$I_{\text{K}}={\tilde{g}}_{\text{K}}n^{4}\left(V_{\text{m}}-E_{\text{K}}\right)$$

where *E*_K_ is the electrochemical-gradient-dependent Nernst potential of K^+^ ions, ${\tilde{g}}_{\text{K}}$ is the single K^+^ channel conductance, and *n* is the Hodgkin–Huxley type K^+^ channel activation variable.

In addition to the above mechanisms, the biophysical model of the myelinated axon featured Na^+^–K^+^ pumps, specific Na^+^ and K^+^ leak currents, extracellular K^+^-dependent swelling of perinodal astrocytes, and longitudinal diffusion of ion species across the different compartments. A detailed description of the model organization, biophysical mechanisms, and modeling techniques is given in Volman and Ng (25, 41).

Injury was imposed uniformly along the model myelinated axon, with all nodes of Ranvier subjected to the same strain. To probe signal propagation along the model myelinated axon, the axon was stimulated at the initial axonal segment (square stimulus; duration, 1 ms; strength, 0.08 nA), and spike amplitude and latency were measured at the penultimate nodal compartment at the other edge (to avoid the boundary effects associated with the ultimate, sealed-end, compartment).

### Neuronal Network Model

Concussion can induce attention deficits of different types: sustained, selective, alternative, and divided (45, 46). Neuronal structures that mediate attention (47, 48) include visual, parietal, frontal, medial temporal, sub-cortical, and reticular areas, with interhemispheric signaling playing an important role (49, 50). Mild traumatic brain injury-induced damage to any of these areas can disrupt attention networks and compromise performance.

A biophysically feasible two-dimensional neuronal network model was developed to link axonal dysfunction of the corpus callosum to neurobehavioral observables representative of clinical outcomes. The model consisted of 6,400 neurons, 80% (5,120 neurons) of which were excitatory pyramidal (PY) neurons and the remaining 20% (1,280 neurons) were fast spiking (FS) interneurons. Gross hemispheric organization was modeled by dividing the model network into two equally sized symmetric sub-networks (representing the two hemispheres). Inside each one of those hemispheric sub-networks, model PY and FS neurons projected and received synaptic contacts from other neurons found within their synaptic footprint (a 10 × 10 region around a neuron). These connections modeled local intra-columnar cortical connectivity. In addition, long-range connections between PY and PY, PY and FS neurons were probabilistically established within the same hemisphere. To model interhemispheric communication through callosal axons between model neurons (both PY and FS neurons), each model neuron established an exact homotopic connection with its counterpart in the opposite hemisphere and a number of loose homotopic connections (with a certain probability) within the footprint of its contralateral counterpart.

The dynamics of PY and FS neurons were described by modified Morris and Lecar (51, 52) and Wang and Buzsaki (53) models, respectively. The synapses between neurons were modeled by α-amino-3-hydroxy-5-methyl-4-isoxazolepropionic acid, *N*-methyl-d-aspartate, and gamma-aminobutyric acid A synaptic currents, with distance-dependent axonal conduction delays. Short-term synaptic depression was modeled as the Tsodyks–Markram type (54); for each synapse, we assumed a phenomenological “synaptic resource” that was reduced after each successful release of synaptic neurotransmitter and recovered exponentially. To model the effect of axonal injury on synaptic transmission, we have adopted and modified the model of Destexhe et al. (55), which prescribes the relation between the presynaptic voltage and the amount of neurotransmitter released. To model attention deficit, a transient (duration, 500 ms) attention-like stimulation was delivered to two symmetrical sub-networks (20 × 20 model neurons, excluding FS neurons) in different “hemispheres,” parameterized as an increase of 100 Hz over the rate of the background stimulation that was administered simultaneously to all model neurons. The attention-like stimulation was applied after the network (driven by the background stimulation) reached a steady state. A detailed description of the model equations, parameters, and analysis methods can be found in Cui et al. (56).

### Neurologic Injury Measure (NIM)

A NIM has been developed for quantifying the extent of cellular-level injury occurring in brain structures and regions. NIM is an internal injury metric and because it represents a fundamental injury quantity (i.e., axonal function), it is independent of species or exposure conditions. NIM quantifies the average degradation of axonal signaling functionality over a region of interest (e.g., the corpus callosum):

(8) $$\text{NIM}=\frac{\sum_{i=1}^{N_{\text{E}}}V_{i}\Delta A_{i}}{\sum_{i=1}^{N_{\text{E}}}V_{i}}$$

where *V_i_* is the volume and Δ*A_i_* is the average reduction in the axonal action potential amplitude, calculated from the biophysical signaling model for the *i*-th element (as defined by the FEM) in the corpus callosum.

### Datasets

The measured head kinematics from three independent and unique data sets used for simulation in the E2E model represented sports exposure, military combat exposure, and NHP concussive head motion. The sports data were the National Football League (NFL) helmet-to-helmet impact data, with six kinematic degrees of freedom (DOF) per player; the dataset comprised 10 concussed and 14 non-concussed players (2, 57). The military combat exposure data came from helmet sensors and were provided by the Joint Trauma Analysis and Prevention of Injury in Combat (JTAPIC). The triaxial linear head kinematics at the center of gravity for 23 concussed and 32 non-concussed subjects were provided. The angular head kinematics for frontal and lateral directions were estimated using the Pellman et al. (2) correlation between translational and rotational acceleration. The outcome of concussion was based on clinical assessment of a state of altered consciousness or loss of consciousness and was determined by the data source. The NHP data were derived from the experimental work of Abel et al. (58). The head acceleration was controlled by a pneumatic piston that produced consistent head motion. The peak rotational acceleration and distance from the centroid of the head to the pivot point were reported in the literature (58) along with some example traces. The source data are no longer available, but Lee et al. (59) proposed a shape function for the rotational acceleration based on published traces. The published peak rotational acceleration and fixed parameters of the apparatus (total angle traversed and angle at which deceleration began) were used for deriving the rotational acceleration traces for the NHP experiments. Injuries of grades 3 and 4, characterized by a brief loss of consciousness and neurological alterations, were classified as concussive, yielding 16 concussed and 13 non-concussed cases. Below grade 3, unconsciousness or lateralized neurological deficits were not observed. NIM values were calculated for each subject and plotted against an outcome of 1 for concussed and 0 for non-concussed. A logistic regression analysis was used for developing a dose-response curve for a general acute concussion outcome.

## Results

### Component Model Analyses

#### Finite Element Model

**Figure 4:**
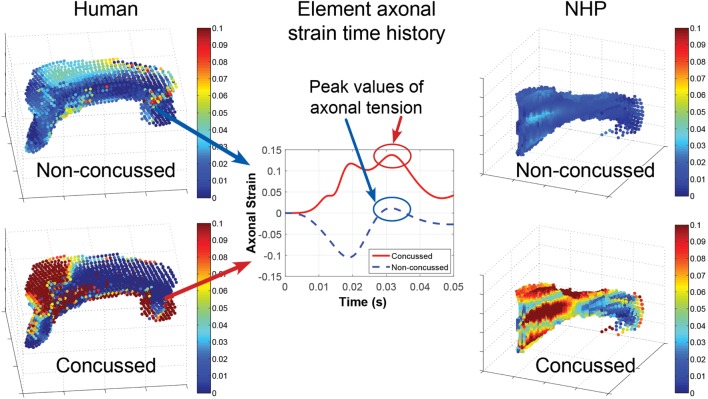
The head finite element models translate head motion into transient and spatial strains in the corpus callosum. In general, the concussed datasets exhibit higher peak axonal strain and strain rates over the corpus callosum compared with non-concussed datasets. Depicted are the maximal tensile axonal strains over all time calculated for each element of the corpus callosum for: a non-concussed and concussed NFL kinematic dataset (left) and a non-concussed and concussed non-human primate (NHP) kinematic dataset (right). An example of the axonal strain time history for the same single element in a concussed and a non-concussed case is depicted in the middle plot. The strain time history is input into the micromechanics model, which calculates strain at the nodes of Ranvier.

The response of the head FEMs was analyzed by examining the corpus callosum axonal strain time histories. The head kinematics from the NFL concussion dataset were applied to the human head FEM. Projection of the resulting corpus callosum tissue strain along the axial direction of the axons yielded patterns of higher overall peak tensile axonal strain as well as strain rates for concussed compared with non-concussed cases. Figure 4 demonstrates that, overall, the maximum axonal tensile strains over all time were higher in the concussed versus non-concussed NFL cases (Figure 4). The same pattern was observed from analysis of the NHP head FEM after applying head kinematics from the NHP data. Maximal axonal strain rates were observed during the relaxing phase of the corpus callosum deformation. These axonal strain time histories (Figure 4) for each element in the corpus callosum were then provided to the axon micromechanics model. The model is comparable to recent FEMs that incorporate axonal directionality in strain calculations with axonal strains lower than maximum principal strains (16, 60).

#### Axon Micromechanics Model

**Figure 5:**
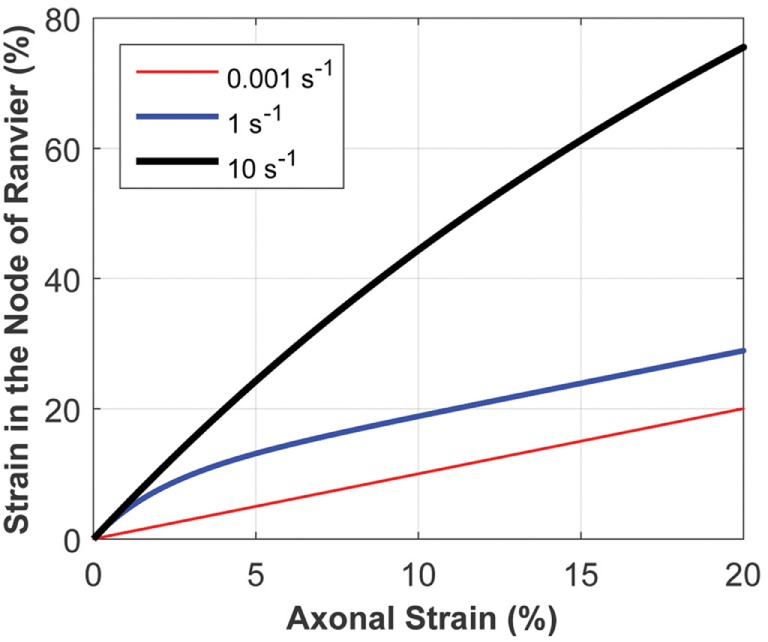
The micromechanics model calculates the strain at the node of Ranvier, given the axonal strain and strain rate. The strain at the node of Ranvier strongly depends on the strain rate, owing to the inherent material behavior of the heterogeneous structure of myelinated axons. This plot demonstrates the relationship between the strain at the node of Ranvier and axonal strain, for a uniform axon diameter in the internode and nodal regions, for varying axonal strain rates.

The micromechanics model accounts for the rate-dependence of a composite structure by mathematically capturing the relatively viscous nature of myelinated regions and the relatively elastic behavior of the underlying axon. To examine the system’s response, the micromechanics model was tested using three different ramp inputs, with physiologically relevant strain rates of 0.001, 1, and 10/s (Figure 5). Strain localized at the model nodes of Ranvier depended on the axonal strain and strain rate, with the latter playing a very significant role. The myelinated regions are more viscoelastic than the underlying elastic axon; thus, high strain rates stiffen the internodes and make them relatively rigid, resulting in strain concentration at the nodes of Ranvier. Conversely, at sufficiently low strain rates, the influence of myelin is weaker, making the strain more evenly distributed throughout the myelinated axon. As shown in Figure 5, the nodal strain can increase more than fourfold in magnitude for a given axonal strain, depending on the axonal strain rate.

The effect of structural variations was also studied. Variations, such as a lower *g*-ratio (increase in the myelin thickness relative to the bare axon diameter), lower *n*-ratio (narrowing of the bare axon diameter in the nodal region), and increased internodal to nodal length ratio, yielded higher nodal strains (not shown). Axons in the human corpus callosum are quite thin, with typical diameters of corpus callosum axons connecting frontal and visual regions (important for executive function and visual attention) being ~1 μm (61). For axons with diameters in this range, the *g*-ratios and *n*-ratios are considered to be constant (62).

#### Coupling the Micromechanics Model with the Biophysical Signaling Model

**Figure 6:**
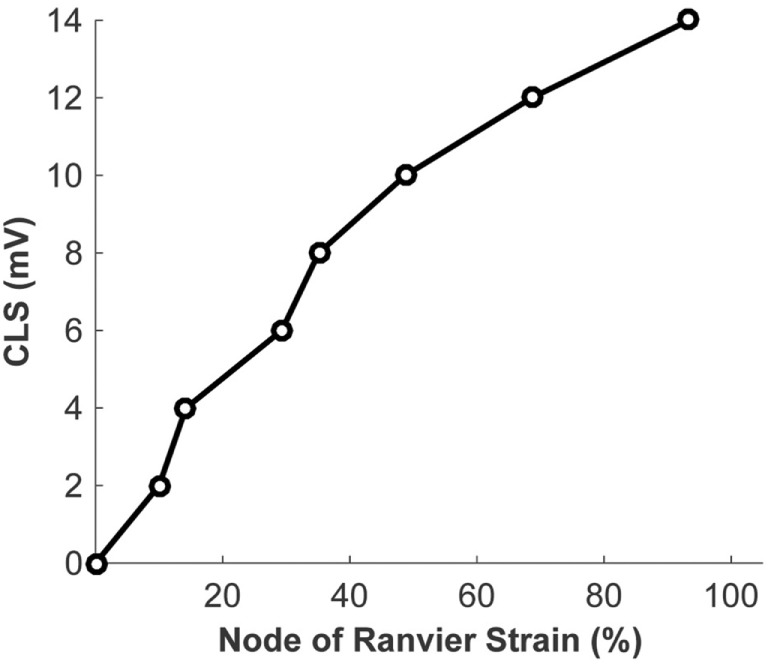
Relationship between the strain at the node of Ranvier and the coupled left shift (CLS) resulting from injury of the nodal voltage-gated Na^+^ channels.

**Figure 7:**
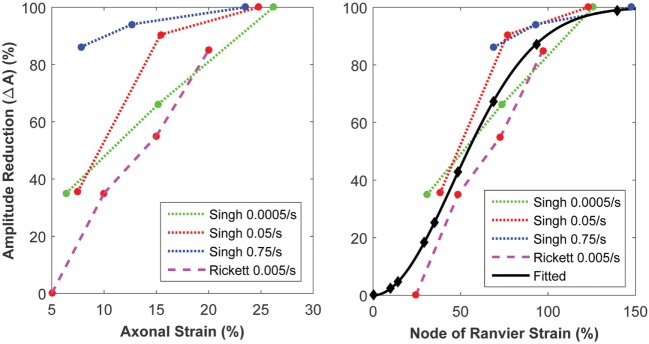
Strain of the node of Ranvier (right) yields a superior correlate of axonal signaling amplitude reduction, compared with the global axonal strain (left).

The biophysical signaling model translates the physical stretching and injury of the axonal nodes of Ranvier into the functional degradation of the axonal signal. The results of nerve stretch injury studies (36, 37) were used to derive the relationship between the CLS of the activation and inactivation voltages of nodal voltage-gated Na^+^ channels and nodal strain (calculated by the micromechanics model), based on the analysis of nerve conduction characteristics for nerves subjected to variable strains and strain rates. A relationship between nodal strain and CLS was derived to account for the effect of nodal strain on alterations in axonal signal propagation (Figure 6). In these studies, the effects of strain and strain rate on neurophysiological functional responses of *in vivo* spinal (36) and *in vitro* sciatic (37) nerves were quantified in terms of changes in compound action potential amplitude and conduction velocity. The applied strains were in the 5–30% range, with strain rates in the 0.0005–0.75/s range. Analysis of the axonal strains and strain rates from the nerve stretch injury studies revealed a general trend toward increasing amplitude reduction with increasing axonal strain. However, when the same data were re-plotted using the strain at the node of Ranvier as a correlate, the variability in the data was greatly reduced (Figure 7).

#### Neuronal Network Model

**Figure 8:**
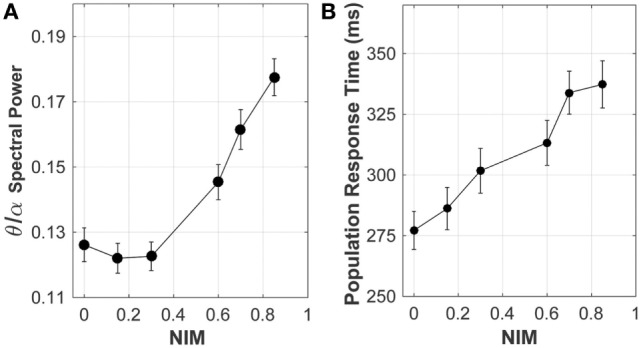
Response of the network model to increasing levels of corpus callosum injury. As the extent of injury increases (i.e., increasing reduction in the axonal spike amplitude, indicated by NIM), both **(A)** the theta/alpha spectral power ratio and **(B)** the population response time (a proxy of reaction time) increase, consistent with clinical observations.

Analysis of the neuronal network output, quantifying the effect of signal degradation of the axons in the corpus callosum on the network dynamics, is summarized in Figure 8. Spectral analysis of the model local field potential, calculated by averaging over membrane potentials of model neurons (63, 64) (sampled from the stimulated areas of the model network), shows that the theta-to-alpha ratio (quantifying the relative “slowing down” of network rhythms) increased with increasing average callosal injury (parameterized by NIM), consistent with results of clinical quantitative electroencephalography (qEEG) analysis of mTBI patients (65, 66).

### E2E Model Analysis

#### Concussion Risk

Logistic regression analysis was used to develop a predictive relationship between two risk correlates (peak linear acceleration (PLA) of the head and NIM) and the probability of concussion. Concussion outcome is variable over a population but is a binary outcome for an individual. Therefore, each data point in the three concussion datasets examined was marked as “1” for a concussed individual and “0” for a non-concussed individual, and a dose–response curve to predict the probability of a general concussion outcome given NIM was developed using logistic regression. It is standard practice to construct a statistical logistic regression from binary data using a logit function:

(9) $$\ln\;\;\left(\frac{p}{1-p}\right)=\beta_{0}+\beta_{1}x$$

where *p* is the probability of concussion and β_0_ and β_1_ are the model’s coefficients (67). A relationship between the risk correlate (i.e., PLA or NIM) and concussion probability was developed using the logit method, where *x* = ln(PLA) or $x=\ln\;\;\left(\frac{\text{NIM}}{1-\text{NIM}}\right)$. The ln(PLA) was used rather than PLA to ensure that *p* = 0 when PLA = 0. Similarly, $\ln\;\;\left(\frac{\text{NIM}}{1-\text{NIM}}\right)$ was used rather than NIM to ensure *p* = 0 when PLA = 0 and *p* = 1 when NIM = 1. The coefficients were estimated using the maximal likelihood estimation.

Logistic regression analysis based on purely kinematic variables (e.g., PLA) showed that the curves developed from the three impact datasets diverge significantly depending on the exposure type and/or species used to build them. Note that the NHP PLA data are scaled to equivalent human injury exposures using the scaling rules, which relied upon physical dimensions, force-time profiles, and mechanical brain response, developed by Stalnaker et al. (68, 69).

**Figure 9:**
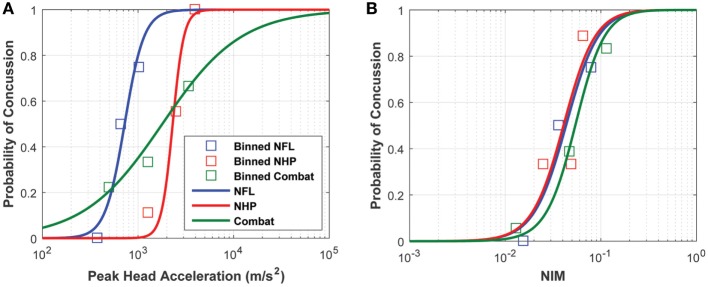
Logistic regressions developed from **(A)** peak linear head acceleration and **(B)** NIM show that NIM, an internal injury measure, produces a tighter probabilistic risk correlate compared with correlates developed from external measures like peak linear head acceleration.

The logistic regressions developed using the sports-type exposure data, combat-type exposure data, and NHP exposure data all overlapped but displayed a great deal of variability. However, the logistic regressions obtained using NIM as the independent variable converged without any treatment of the NIM (Figure 9). For visualization purposes, an average injury probability was plotted by binning the concussion outcomes over equally spaced intervals along the *x* axis.

**Figure 10:**
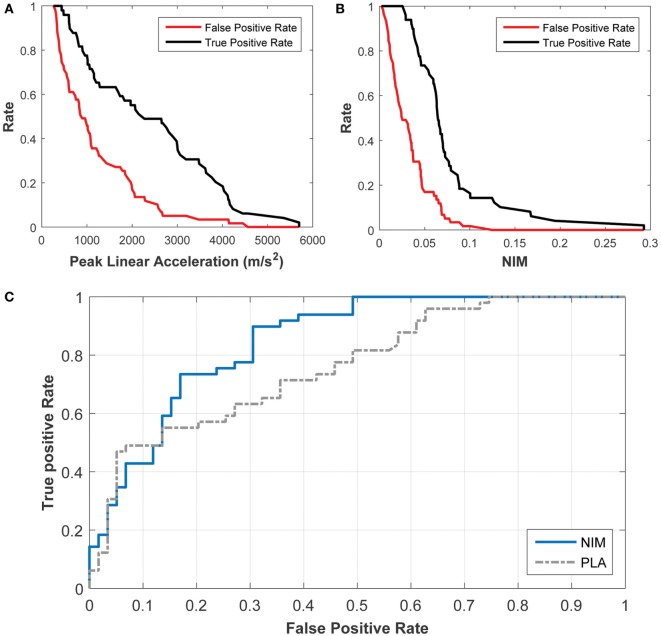
Receiver Operating Characteristic (ROC) analysis of the probabilistic concussion models developed based on NIM, compared with those developed based on peak linear head acceleration. The rate of true positives (black) and the rate of false positives (red) can be used to determine the rate for a particular injury threshold using **(A)** the peak linear acceleration correlate and **(B)** the NIM correlate. **(C)** The ROC curve shows that 90% of true positives (sensitivity) correspond to 30% of false positives (specificity) for NIM, while 90% of true positives correspond to 60% of false positives for peak linear head acceleration.

A receiver operating characteristic (ROC) statistical analysis of the logistic regression models shows that NIM provides a better sensitivity (fraction of true positives) and specificity (fraction of true negatives) compared with PLA (Figure 10). For example, using NIM, the fraction of true positives is 90% while the fraction of false positives (e.g., 1 − specificity) is 30%. This is a significant improvement over the PLA correlate, in which the fraction of true positives of 90% is associated with a 61% fraction of false positives. This type of analysis shows that the logistic regression can be used to determine the trade-offs for setting a threshold.

The area under the ROC (AUROC) was also calculated for both logistic regression models. An AUROC of 1 implies that the model is accurate 100% of the time while an AUROC of 0.5 implies that the probability of accuracy is 50% (or a random guess). The AUROC for the NIM regression is 0.85 whereas the AUC for PLA is 0.76.

To assess how well logistic regression fits the data, the Hosmer–Lemeshow test statistic was applied:

(10) $$H=\sum_{g=1}^{G}\frac{{\left(O_{1g}-E_{1g}\right)}^{2}}{N_{g}\pi_{g}\left(1-\pi_{g}\right)}$$

where *O*_1*g*_ corresponds to the number of the observed “1” events, *E*_1*g*_ corresponds to the number of the expected “1” events, *N_g_* is the overall number of observations, π*_g_* is the predicted risk for the *n*-th risk decile group, and *G* is the number of groups (*G* = 10 in this study). The statistic was approximated by the chi-square distribution with *G*-2 DOF, and a *p*-value was calculated. The fits to the data were nearly the same for PLA and NIM, with *H* of 7.92 and 7.96, respectively. The *p*-value was 0.44 for PLA and NIM.

#### Neurobehavioral Outcome

Brain rhythms in the alpha (8–12 Hz) and beta (13–30 Hz) frequency bands have been linked to performance on cognitive tasks and memory (70). In addition, beta rhythm has been associated with top-down attention (71) and long-range inter-areal interaction between different cortical areas, with longer associated axonal conduction delays (72, 73). The intact model neuronal network exhibited low neuronal spiking rates, with alpha band (8–12 Hz) collective rhythm in the resting state and beta rhythm dominance during the network stimulation (56).

Altered cortical dynamics owing to the corpus callosum injury (quantified by NIM) were comparable to the existing qEEG data (Figure 8). In the clinical setting, a relative increase in the theta power and a relative reduction in the alpha power (termed “slowing down of rhythms”) indicate cognitive and neuropsychological deficits (74), such as attention deficit, which are often related to an increased reaction time observed in neurobehavioral testing of mTBI subjects. In model networks with injured callosal axons, the maximal response to attention-like stimuli developed at a later time compared with the intact model network (Figure 8). The population response time, defined as the time from the stimulation to development of maximal response, was used as a proxy of reaction time that is often measured in neurobehavioral attention tests. The increase in the population response time with increasing the injury severity (measured by NIM) (Figure 8) is consistent with clinical reports of longer reaction time in mTBI patients, compared with healthy controls (75–78). A more comprehensive analysis revealed that the population response time depended on the injury-induced reduction in axonal spike amplitude, rather than on injury-induced changes in axonal conduction time (56).

## Discussion

A quantitative E2E model that integrates the fundamental mechanical, physiological, and neurological processes associated with traumatic brain injury has been developed, and is the first comprehensive model of its kind. The designed model aims to distill the major processes involved in producing concussive outcomes, from traumatic head motion to neurological injury, by incorporating the most prominent injury mechanisms, from macroscopic tissue mechanics to cellular scale processes. Such a multi-scale approach toward a more biofidelic concussion model has been identified as a necessary step toward more accurately identifying those individuals who may have been injured, guiding the development of personal protective equipment, and guiding the development of current and future monitoring technologies (33, 79).

A mechanistic model can also better utilize animal data. For obvious ethical reasons, a majority of concussion research is conducted on animals rather than on humans. While animal models have provided insight into a range of injury mechanisms, from head loading to axonal injury, kinematic measures such as PLA require scaling from animal to humans. Often, such a scaling involves non-dimensional analysis of several parameters (e.g., head acceleration, duration of the head acceleration, impact velocity, average skull thickness, average bone thickness) and is an imprecise technique. Because the E2E model is a mechanism-driven model, it does not suffer from the inherent limitations that external-based correlates or species-derived correlates face. The value of this modeling approach is demonstrated through its ability to unify concussion datasets gathered from a range of conditions, including a sports environment, a combat environment, and data gathered from animals (e.g., NHPs), without the need for scaling.

The framework driving this modeling concept starts from the observation that concussion can be correlated to disruption of neurological tissues in areas of the brain affected by injury. White matter tracts, and in particular the corpus callosum, have been identified in DTI studies as having reduced fractional anisotropy, implying reduced structural integrity of these regions following injury (collectively characterized as diffuse axonal injury) (80). Deformation of brain tissue following traumatic head motion is driven by the internal biomechanics. The extent of axonal injury depends on the magnitude, rate, and direction of deformation. In the present work, we focused on the axons of the corpus callosum for injury quantification because: (1) imaging studies suggest reduced integrity of corpus callosum axons following injury (21, 81); (2) FEM simulations predict that head kinematics associated with concussive outcomes yield the highest strain concentration in the corpus callosum (10–13); and (3) the corpus callosum plays an important role in interhemispheric communications.

### Fundamental Processes Associated with Concussion Injury

The E2E model starts with head kinematics as input into the head FEMs, which capture anatomical details, such as the boundary conditions between the skull and brain as well as the segmentation of structures that comprise the brain; these features are necessary for accurate modeling of the internal brain biomechanics. Owing to the frictionless sliding between the dura and brain components and the low shear modulus of the brain tissue, significant initial motion and twisting of the corpus callosum in the direction opposite to that of the applied head motion is observed. While the attachment of the outer edges of the falx cerebri and tentorium cerebelli to the dura impedes the brain motion to some extent, there is still significant motion of the corpus callosum relative to its original location in the brain, even for the non-concussed cases. Our model is consistent with other FEMs analyzing NFL head impact data, and suggests that largest strains are observed after the primary head acceleration, with large strains observed in the corpus callosum (10, 12). Additionally, the inclusion of axial DTI direction in the analysis of axonal response to impacts more accurately portrays deformation associated with injury outcomes and becomes increasingly important in analyzing impact direction sensitivity in relationship to injury severity and location (16, 60). The quantification of strain along the axons is a key element in the E2E model; strains that are normal to the axonal axial direction will have a minimal effect, while strains in the axonal axial direction will produce a maximal stretch. Future extensions of this model will expand axonal injury quantification to other white matter areas as well as exploration of injury from compression (82) and shearing forces (3, 83).

The literature suggests that the nodes of Ranvier are susceptible to stretch injury (22). The high cholesterol content of myelin makes its viscoelastic response very different (relatively stiffer especially at high strain rates) from that of the bare axonal bilayer (35), likely resulting in regions (e.g., node of Ranvier) of strain concentration at high strain rates. The micromechanics model was built to incorporate the physics of the axonal structure. Physical elongation of nodes of Ranvier in response to axonal stretching has been confirmed in histological studies (22). As shown in Figure 5, the micromechanics model suggests that the nodal strain strongly depends on the axonal strain rate. Animal models (*in vitro* and *in vivo*) of nerve stretch injury confirm that strain and strain rate significantly determine injury-induced nerve function alterations (36, 37). The micromechanics model quantifies the effects of these parameters on the strain localized along the axon, particularly at the nodes of Ranvier. The model demonstrates a stronger correlation between the nerve function and the strain at the nodes of Ranvier, compared with the global axonal strain (Figure 7), thus confirming experimental observations that a high strain applied at a low rate yields functional decrements that are similar to those obtained after applying a low strain at a high rate (36). This highlights the importance of considering both strain and strain rate when making injury predictions.

The biophysical signaling model quantitatively explains the mechanism by which mechanical strain of the node of Ranvier leads to impaired signal transmission along the axon. *In vitro* experiments have shown that stretch injury of tetrodotoxin-sensitive Na^+^ channels, known to be concentrated at nodes of Ranvier (84), manifests itself as a CLS of the channel activation and inactivation voltages (23), thus altering the channel kinetics and likely profoundly affecting the ability of the nodal membrane to support action potentials. Yuen et al. (85) further demonstrated upregulation of Na^+^ channel protein expression following stretch injury, thus further suggesting that Na^+^ channels might be injured during stretch.

The E2E model quantifies physical tissue and cellular deformation and injury at the node of Ranvier resulting from head kinematics. However, brain injury can be mediated by a number of pathways. Studies have demonstrated that injurious pressure gradients can result from linear acceleration (86). For blast-induced mTBI, additional mechanisms resulting from primary blast exposure have been hypothesized, including transmission of the pressure wave through the skull (87, 88), cavitation (89), skull flexure (90), pressure-driven head motion (91, 92), and blood surge from the body to the head as a result of torso compression from blast to the thorax (93). The primary injury mechanism is likely dependent on the specific exposure characteristics. This model demonstrates a mechanism of injury from violent head motion arising from an impact to the head in which resulting strain and strain rates are injurious to the nodes of Ranvier of myelinated axons in the corpus callosum.

The present model assumes the nodes of Ranvier in myelinated white matter axons as the primary locus of injury. Although this assumption is backed by some imaging studies, as well as *in vitro* and mechanistic models of axonal injury, it is clear that, in reality, injury is not limited to nodes of Ranvier. On the microscopic level of axonal response to stretch, we have identified partial demyelination of paranodal and juxtaparanodal compartments (flanking the nodes of Ranvier and housing a high density of voltage-gated K^+^ channels) as an additional mode of injury (41, 42). Consistent with the findings of *in vitro* models (22) such injury-induced demyelination persistently altered axonal excitability, which could potentially induce pathological outcomes. Damage to white matter axons can also be inflicted following limited energy supply and metabolic injury (94), introducing the possibility of mechanistically induced micro-vascular injury (e.g., manifested as micro-bleeds), which has not been accounted for by our present model. In addition, axonal excitability can be altered directly by stretch-induced mechanoporation and increased membrane leakiness (95). This pathway was not addressed in the present model but can be introduced as shown recently (96).

*In vitro* models show that neurons and glial cells (in particular astrocytes) also respond to mechanical stimuli (97–100), and imaging studies often show that injury extends to gray matter (101–103), suggesting that the later is also affected in mTBI. However, it remains unclear whether mTBI-induced micromechanical strains on gray matter neurons and astrocytes are comparable to the strains used in the published *in vitro* models. The geometry of gray matter is relatively isotropic, compared with the relatively anisotropic axonal geometry. In addition, assessment of injury sequelae (usually not performed immediately after injury) may capture secondary processes (e.g., injury-induced homeostatic regulation in neurons and reactive gliosis in astrocytes), which precludes the precise assessment of primary effects (associated with the mechanistic injury *per se*). Although our present model only focuses on white matter axonal injury at the nodes of Ranvier, the E2E modeling approach can be used to extend the model to couple injury-related head kinematics to microscopic strains in gray matter. If successful, this approach will enable to more clearly delineate the different brain structures affected in mTBI.

### Neurologic Injury Measure as the Internal Injury Correlate

The outcome of concussion depends on many individual physiological factors that are not captured by the head motion alone. Consequently, a distribution of outcomes is observed for the same head motion, thus necessitating a probabilistic risk assessment. The E2E model, quantifying the average injury-induced signal degradation in myelinated axons of the corpus callosum, correlates NIM with two different types of outcomes. Because axons are a basic functional unit of the brain for all species, it follows that axonal dysfunction, quantified in our model in terms of the reduction in the action potential propagation over a region, is a primary candidate for an internal injury correlate. The first correlation derived from the E2E model is tied to a general concussion risk outcome. The second correlation goes beyond this and links a localized brain injury to observed concussive symptoms, which may have more practical implications for understanding the return-to-play criteria, the impact of concussion on day-to-day functionality, and the temporal evolution of symptoms.

For the first correlation, the logistic regressions that were developed based on PLA and NIM were characterized in terms of: (1) the goodness of fit and (2) the model’s predictive ability. Goodness of fit was analyzed using the Hosmer–Lemeshow tests. Both PLA- and NIM-based logistic regressions performed equally well, yielding similar chi-squared and *p*-values. The logistic regressions fit each of the datasets well. Yet, goodness of fit is not indicative of the accuracy of model predictions. To assess this accuracy for a continuously varying threshold, a ROC curve was plotted to demonstrate the relationship between the rate of true positives and the rate of false positives (Figure 10), and the AUROC confirmed that the NIM has greater predictive ability compared with the PLA. For most of the spectrum, NIM exhibited a higher ratio of true positives to false positives compared with PLA, implying that the NIM can better predict the risk of concussion. Figure 10 shows the trade-offs for selecting varying thresholds of concussion risk. These statistics reveal the power of modeling the mechanism of injury and quantifying an internal correlate, which implicitly accounts for a wide range of external factors and is species independent.

**Figure 11:**
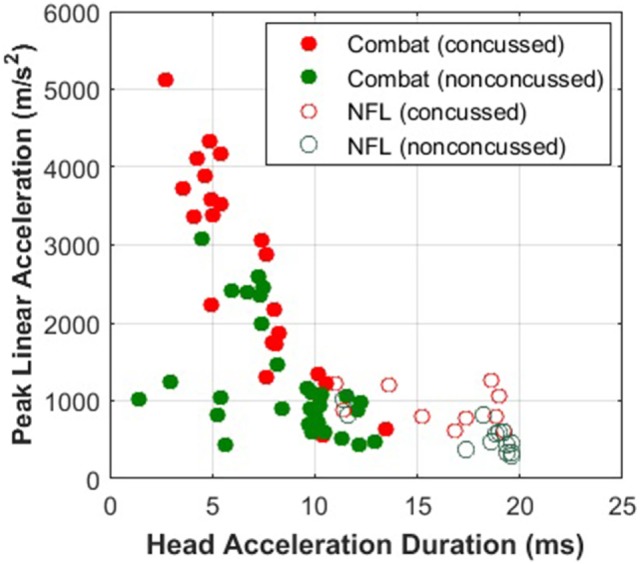
Military impacts are shorter and more violent (higher peak head acceleration) than sports impacts. The Combat data was provided by Joint Trauma Analysis and Prevention of Injury in Combat (JTAPIC). The National Football League (NFL) data was extracted from Pellman et al. (2). Concussion thresholds drawn to combat impacts would not be applicable to thresholds drawn to sports impacts.

The need for modeling an internal injury correlate becomes clearer when examining concussion data from different types of exposures. PLA has been the quantity of choice for concussion correlates owing to the ease of measurement, but, as shown in Figure 11, the injury thresholds may only hold for the conditions under which the data are collected. Peak rotational acceleration has also been explored as a correlate, but it suffers from the same limitations as PLA (104). A number of computational models have been developed to provide insights into the response of internal tissue biomechanics, with the assumption that mechanical damage leads to functional disruption (32, 33). The E2E model completes the pathway, connecting mechanical perturbations to signaling alterations on the axonal level.

The second correlation builds upon the quantification of callosal axon disruption and its link to specific neurobehavioral symptoms via the neuronal network model component of the E2E model. The corpus callosum is a highly organized body of axons, the majority of which are myelinated, connecting the left and right hemispheres. Thus, this structure plays a primary role in integrating motor, sensory, and cognitive processing in the two hemispheres (105–108), and likely significantly shapes some well-known neurobehavioral sequelae of mTBI, such as altered top-down attention (75–78), reaction time (75–78), and working memory (109).

The model neuronal network dynamics were affected by corpus callosum injury. These injury-induced changes included: (1) “slowing down” of the network rhythms, manifested as an increased resting-state theta-to-alpha power ratio (Figure 8), (2) reduced response to attention-like stimulation, manifested as a reduced spectral power of collective activity (data not shown), and (3) increased population response time in response to stimulation (Figure 8). Importantly, these changes were not only consistent with clinical data (65, 66, 110–112) but were also positively correlated with corpus callosum injury severity. Clinical data suggest that working memory (the ability of cortical circuitry to transiently “remember” the stimulation after its cessation) is impaired after mTBI (111, 113). Our preliminary results on network modeling of injury-induced working memory dysfunction verify this; working memory (parameterized as the duration of post-stimulus persistent activity) is affected by the corpus callosum injury, in a manner that depends on the injury severity (114).

Reaction time has been used as one of the few neurobehavioral indices for quantifying mTBI sequelae (75, 76, 78, 109) and has been proposed as prognostic utility for acute mTBI (115, 116). In our neuronal network model, reaction time was approximated by the population response time, which was defined as the time at which the model network exhibited maximal response to attention-like stimulation. Our present result of increased population response time (Figure 8) is in a qualitative agreement with previously published clinical results of increased reaction time for mTBI patients engaged in cognitive and behavioral tasks (75, 78, 117–119). Most importantly, the dose-dependence of the population response time on callosal injury severity in our model is consistent with clinical observations of longer reaction time for mTBI patients with injured corpus callosum (112), thus supporting the proposal of using reaction time as an objective biomarker for mTBI (115, 116).

### Application of the E2E Model

The application of the E2E model, based on the implementation of fundamental physiological and neurological processes associated with concussion, significantly improves upon the commonly used PLA correlative for the prediction of concussion from violent head motion and, thus, has an immediate application in the growing field of head-worn sensors that screen for possible mTBI. Using the model insights, concussed individuals can be more objectively identified in near real time, by integrating a simplified version of the E2E algorithm into the sensor microprocessor or in post-exposure data analysis after data download. Early identification of those injured is critical, because rapid treatment can mitigate chronic mTBI outcomes and reduce lifetime medical costs (120). Furthermore, the E2E model can be applied to add a mechanistic interpretation of the injury outcome to the copious amount of data being collected by a number of ongoing studies that are focused on characterizing medical outcomes associated with head impact intensity, impact frequency, and impact distribution measured from head-worn sensors in various sports (104, 121, 122) and military environments (123). In addition, understanding the connection between head acceleration details and physiological outcomes will allow equipment designers to maximize the protective nature of gear and exposures. Last, our model suggests that the outcome of concussion, based upon external correlates, varies considerably with the impact characteristics, thus making it difficult to establish a universal set of safety standards and methodology for evaluating operational trade-offs; whereas the NIM is able to unify data from a range of exposure conditions. By understanding the mechanism of injury, the E2E model can also be used to improve protection designs, set safety exposure standards, and guide future monitoring technology through sensor placement, interpretation, requirements, and evaluation.

The E2E model represents the first comprehensive mechanistic-based approach that quantitatively links gross head motion to neurological outcomes. However, several limitations should be recognized. First, the component models are based upon published data, which are limited in certain aspects; thus, additional validation datasets would be helpful for improving the models. For example, the validation of the FEMs would benefit from more rigorous evaluation. Additionally, anatomic features, such as the spinal cord and CSF, are not explicitly modeled in the FEMs, but rather accounted for with boundary conditions and contact treatments that aim to reproduce the physical response of such features. These simplifications reduce the accuracy of the response at these locations in the model (e.g., outer regions of the cortex) and likely have minimal influence on the corpus callosum response. Cellular-level aspects, such as localized strains calculated by the micromechanics model and implemented processes of axonal signaling models, need to be addressed with care and are continuously explored as new experimental data become available. Second, the component models are relatively simple by design so that they are able to capture the relevant injury mechanisms without overly complicated detail. With the E2E framework in place, it is reasonable to augment the detail and complexity of components models by including more anatomical, physiological, and neurological details. Third, the logistic regressions were built using outcomes that were categorized as concussed or non-concussed. The two human datasets provided the diagnosis of concussion as determined by the data source. Concussion was based on clinical assessment of a state of altered consciousness or loss of consciousness. A similar criterion was used for the NHP dataset for determining the occurrence of concussion. As the definition of concussion is refined, the logistic regressions will also be refined. Last, a larger concussion dataset would improve the accuracy (i.e., sensitivity and specificity) of the logistic regressions. Despite these limitations, the E2E model has been demonstrated to be a significant improvement to the current external-based correlative concussion models.

The framework of the E2E model has been established and validated, providing a strong foundation that can be extended to several areas, beyond the prediction of acute concussion risk and the neurobehavioral consequence of increased reaction time. First, injury of white matter tracts outside of the corpus callosum, focal injury, and gray matter injury can be readily incorporated. Tissue injury from compression and shearing forces should also be studied. On the cellular level, consideration of metabolic injury, stretch-induced mechanoporation, and glial response should be incorporated. Second, the model can be extended to assessment of a range of neurobehavioral consequences, which offers practical insight into the effects of concussion on day-to-day tasks. Third, the model can be extended to better understand the effects and outcome from accumulation of sub-concussive head injuries, which is an area of growing concern, particularly for the sports and military training arenas (124). Recovery is another aspect that needs to be incorporated into the model to more accurately track cumulative injuries; in addition, this aspect is critical for understanding the return-to-play criteria in the sports field. Fourth, the effect of more violent head impacts can be explored by incorporating primary demyelination and secondary pathological outcomes (41). Last, the model can be expanded to explore the compounding effects of operational stressors (e.g., hypoxia, physical exertion, and sleep deficit).

## Conclusion

The E2E model is the first to establish a quantitative framework linking head motion to neurological outcomes by linking the gross mechanics of motion to key pathophysiological processes that result in injury. In this model, tissue response from head motion is translated into strain of myelinated axons of the corpus callosum. The model demonstrates that the node of Ranvier is particularly susceptible to high strain rates, thus providing insight into why violent head motions, which produce both high strains and strain rates, are injurious. Physical straining of the nodal voltage-gated Na^+^ channels results in a CLS shift, thus producing functional alterations in axon signaling. The NIM, or the average reduction in the axonal signaling amplitude, is calculated and used as the internal injury correlate. By using neurologically based quantities rather than external kinematics, the E2E model is able to unify concussion data across a range of exposure conditions and species with greater sensitivity and specificity compared with correlates developed from external-based measures. This mechanism-based model is a significant and necessary advancement over current empirically derived correlates, which are valid for a narrow range of conditions and do not offer a pathway for understanding more complex injury patterns. The E2E model extends beyond being a robust predictor of the risk of concussion to provide models of the neuronal network and injury of brain structures that can be linked to mTBI sequelae, offering insight into how brain injury can be related to clinically observed outcomes. The E2E model provides a strong foundation for extending the analysis to assessment of a range of neurobehavioral consequences, accumulation of sub-concussive head injuries, and inclusion of compounding effects of operational factors on clinically observed symptoms.

## Author Contributions

LN is the PI of this work and oversaw the integration of the component models into the end-to-end model. She developed the research plan, identified key tasks, and focused the group on successfully accomplishing the overall objective. VV is the lead neurophysiological modeler and was primarily in charge of the development, validation, and analysis of the axon biosignaling model. He also oversaw the work of JC in the development of the neuronal network model. MG is the lead finite element modeler and was in charge of the development, validation, and analysis of the human finite element model. She provided guidance in the development and analysis of the non-human primate model. DS developed, validated, and analyzed results from the non-human primate finite element model. PP developed, validated, and analyzed the axon micromechanics model to quantify strain at the node of Ranvier from axonal strain calculated from the finite element model. JC developed, validated, and analyzed the results from the neuronal network model for the prediction of neurobehavioral outcomes from axonal injury. JS is the co-PI and helped in the development of the overall approach.

## Conflict of Interest Statement

The authors declare that the research was conducted in the absence of any commercial or financial relationships that could be construed as a potential conflict of interest.

## Footnotes

^1^https://zygotebody.com.

^2^https://www.nitrc.org.
